# Cytokine Profile in Human Eyes: Contribution of a New Cytokine Combination for Differential Diagnosis between Intraocular Lymphoma or Uveitis

**DOI:** 10.1371/journal.pone.0052385

**Published:** 2013-02-06

**Authors:** Sylvain Fisson, Hanane Ouakrim, Valérie Touitou, Sylvie Baudet, Rym Ben Abdelwahed, Sabrina Donnou, Amine Miloudi, Claire Galand, Bahram Bodaghi, Phuc LeHoang, Martine Brissard, Magali Le Garff-Tavernier, Wolf Herman Fridman, Catherine Sautès-Fridman, Nathalie Cassoux, Hélène Merle-Béral

**Affiliations:** 1 Institut National de la Santé et de la Recherche Médicale, UMRS872, Centre de Recherche des Cordeliers, Paris, France; 2 Université Pierre et Marie Curie-Paris 6, UMRS 872, Paris, France; 3 Université Paris Descartes, UMRS 872, Paris, France; 4 Généthon, Evry, France; 5 Institut National de la Santé et de la Recherche Médicale, U951, Evry, France; 6 Université d'Evry Val d'Essonne, UMRS 951, Evry, France; 7 Service d'Ophtalmologie, Hôpital de la Pitié-Salpêtrière, AP-HP, Paris, France; 8 Service d'Hématologie Biologique, Hôpital de la Pitié-Salpêtrière, AP-HP, Paris, France; 9 Service d'Onco-ophtalmologie, Département d'Oncologie Chirurgicale, Institut Curie, Paris, France; Massachusetts Eye & Ear Infirmary, Harvard Medical School, United States of America

## Abstract

Primary intraocular lymphoma (PIOL), also called primary vitreoretinal lymphomas, often masquerades as uveitis. This misdiagnosis can result in subsequent brain involvement and oculocerebral lymphoma (OCL). In this study, we sought to characterize the helper T-cell type 1 (Th1)/Th2 cytokine profile in vitreous samples from patients with PIOL, OCL, uveitis and controls with non-inflammatory disease. Vitreous and aqueous humor samples from 87 patients with PIOL (n = 30), OCL (n = 12), uveitis (n = 34), and retinal detachment (RD) without hemorrhage (n = 11) were analyzed and their concentrations of interleukin (IL)-2, IL-4, IL-6, IL-10, interferon (IFN)-γ, and tumor necrosis factor (TNF)-α were determined by flow cytometric bead arrays (CBA). The IL-10 levels determined by CBA were compared with those by ELISA. IL-10 concentrations measured by CBA and ELISA were highly correlated. IL-2, IL-4, and TNFα were not detected in any sample. The only cytokine detected at a significant level in samples from RD vitreous was IL-6. The IL-10/IL-6 ratio, as previously reported, was slightly higher in PIOL than in uveitis samples, but not for all patients. Cytokine profiles from PIOL and OCL samples did not differ. The combination of the IL-10/IL-6 and IL-10/IFNγ ratios was highly informative for discriminating PIOL/OCL from uveitis samples and for therapeutic follow up of PIOL. This strategy might be very helpful as an initial screening to rule out PIOL in patients thought to have uveitis.

## Introduction

Primary intraocular lymphoma (PIOL), also called primary vitreoretinal lymphomas [1], is a subset of primary central nervous system lymphoma (PCNSL) that initially presents in the eye, with or without simultaneous CNS involvement [2], [3]. Most PIOL cases are related to high-grade extranodal non-Hodgkin, diffuse large B-cell lymphomas. A rapid definitive diagnosis is required for appropriate treatment to prevent the substantially worse prognosis associated with the spread of the disease to the brain. PIOL often masquerades as chronic uveitis and thus remains difficult to diagnose: a high degree of suspicion is required before testing [4], [5]. A high level of IL-10 in pure vitreous or aqueous humor samples or an IL-10/IL-6 ratio greater than 1 in diluted or undiluted samples is considered indirect evidence requiring further diagnostic testing [6], [7]. The exact cutoff for the IL-10 concentration or IL-10/IL-6 ratio may vary between laboratories, mainly due to differences in methods and conditions of sample harvesting and storage, techniques, and manufacturers of equipment and supplies, as well as the dilution (known or unknown) of the vitreous samples and the laboratory's own experience. These factors result in false-positive (11%) and false-negative (23 to 30%) PIOL diagnoses [6], [8], [9], [10]. Because no systematic cytological analysis strategies currently discriminate between PIOL and uveitis in patients [11], a better characterization of the molecular microenvironment seems essential to identify new cytokine combinations as diagnostic markers. Accordingly, our aim here was first to compare the reference ELISA technique [6] to a multiplex-based cytometric bead array (CBA) technique that enables the simultaneous analysis of several cytokines without sample volume limitations, as only 25 to 50 µL is needed. Recombinant human IL-10 was used to compare the accuracy, sensitivity, and range of concentration analysis of these techniques, and IL-10 was measured in frozen human samples to confirm the robustness of both techniques. In the second part of this work, we analyzed the Th1/Th2 cytokine profile, including IL-10, IL-6 and IFNγ, in vitreous and aqueous humor samples from patients with PIOL, oculocerebral lymphoma (OCL), uveitis, and retinal detachment (as controls). Cytokine ratios from the different groups of samples were compared and allowed us to define a new strategy for discriminating patients with PIOL or OCL from those with uveitis.

## Patients and Methods

### Patients and samples

Ethics Statement: this study was performed in accordance with the Declaration of Helsinki. The patients, who were recruited from the Ophthalmology department of the Pitié-Salpêtrière Hospital (Paris), all provided written informed consent after the nature of the study had been fully explained to them. The Pitié-Salpêtrière hospital review board approved the study. All data were treated confidentially.

The results involving vitreous samples included 60 patients (Table 1), distributed in four groups. The first comprised 17 patients with PIOL, the second 9 patients with OCL diagnosed by cytologic analysis of the vitreous and positive MRI for OCL, the third 23 patients with diagnosed uveitis, and the last (negative control) group, 11 patients with non-hemorrhagic retinal detachment. Vitreous humor specimens were obtained through standard three-port pars plana vitrectomy, as previously described [12]. Tissue culture medium (balanced salt solution, BSS) enriched with 10% fetal calf serum was added to the collection chamber to improve cell viability. An initial 250 µL specimen of pure vitreous humor was collected separately into a microtube for IL-10 quantification only; the remainder was diluted into BSS supplemented with 10% fetal calf serum, and 2 mL of this preparation were delivered immediately, in a syringe, to the Hematology Laboratory. The vitrectomy cutter was maintained within the vitreous humor to obtain a dilute specimen with minimal disruption. The remainder of the diluted vitreous humor specimen, about 20–30 mL, was harvested in a collection cassette and immediately processed for cytological and immunocytochemical analyses.

**Table 1:** Summary of patient characteristics at diagnosis.

Tissue	Final diagnosis	Number	Age	Sex
			Min-max (median)	^§^M/^‖‖^F
**Vitreous**	#PIOL	17	37–89 (75)	5/12
	^†^OCL	9	45–82 (70)	1/8
	Uveitis	23	6–84 (70)	12/11
	^‡^RD	11	54–81 (73)	4/7
**Aqueous Humor**	#PIOL	11	51–88 (71)	4/7
	^†^OCL	3	66–72 (68)	2/1
	Uveitis	11	46–89 (73)	5/6

# PIOL, Primary intraocular lymphoma; ^†^OCL, Oculocerebral lymphoma; ^‡^RD, Retinal detachment; ^§^M, male; ^‖‖^F, female.

After an anterior chamber puncture during or before surgery, 100 µL of aqueous humor was taken from 27 patients for cytokine measurement. If performed before vitrectomy, the anterior chamber puncture was done under topical anesthesia with a 30-gauge needle in the operating theater with a surgical microscope. If it was performed during the surgery or vitrectomy, the patient was under general or local anesthesia. Patients analyzed at diagnosis included a first group of 11 patients with PIOL, a second of 3 patients with OCL, and a third of 11 patients with uveitis. Kinetic analyses were performed for two other patients (patients #1 and #2), after a recurrence; both were treated intravitreously with methotrexate.

### Cytokine assays

Samples were stored at −80°C, and cytokine assays were performed on freshly thawed samples. IL-10 concentrations were determined from minimum 100 µL vitreous samples, with a standard quantifiable sandwich enzyme immunoassay technique (Quantikine®; RD Systems, Abingdon, UK), as previously described [13].

For cytokine measurements by the cytometric bead array (CBA) technique, samples were assayed for IL-2, IL-4, IL-6, IL-10, TNFα, and IFNγ (human Th1/Th2 CBA kit; BD Biosciences), according to the manufacturer's recommendations. Briefly, six capture bead populations with distinct fluorescence intensities and coated with cytokine-specific capture antibodies were mixed together in equal volumes: 50 µL of each sample and 50 µL of PE-conjugated detection antibodies were added to 50 µL of mixed-bead populations. The mixture was incubated for 3 hours at room temperature in the dark to form sandwich complexes. The beads were then washed with wash buffer, and data acquired with an LSR II flow cytometer (BD Biosciences). FACSDiva and FCAP software (BD Biosciences) were used for the analyses. When a value was negative, the theoretical limit of detection value was used for Log calculations.

### Statistical analysis

The PIOL, OCL, and uveitis groups were compared by the Mann-Whitney non-parametric statistic test or the Student t-test. *P*<0.05 was considered significant.

## Results

### IL-10 concentrations measured by CBA and correlation with ELISA

**Figure 1:**
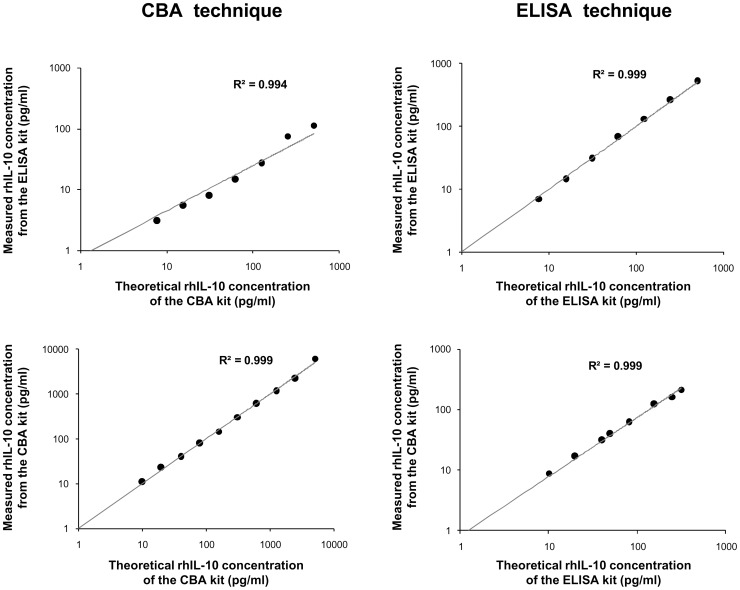
Correlation of recombinant human IL-10 concentrations as measured by CBA and by ELISA. Recombinant human IL-10 (rhIL-10) cytokines provided by the CBA kit (lower graphs) and the ELISA kit (upper graphs) were measured separately by CBA (left graphs) and ELISA (right graphs) techniques. This figure represents one of four experiments.

**Figure 2:**
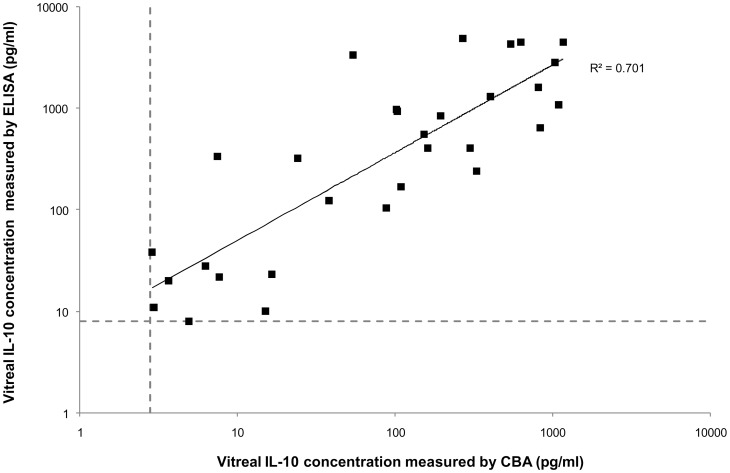
Correlation of vitreal IL-10 concentrations measured by CBA and by ELISA. IL-10 concentration from 30 frozen vitreous samples (13 PIOL, 8 OCL and 9 uveitis patients) were analyzed at the same time. The horizontal and vertical dashed lines indicate the sensitivity thresholds for ELISA (8 pg/ml) and CBA (2.8 pg/ml), respectively.

High levels of IL-10, usually measured by ELISA within pure vitreous or aqueous humor samples, are considered indirect evidence of a PIOL diagnosis [6]. Before using the CBA multiplex technology to analyze the Th1/Th2 cytokine profiles of PIOL, OCL, and uveitis patients, we compared the IL-10 results produced by ELISA and CBA. As Figure 1 shows, measurement by these two different techniques yielded IL-10 concentrations that were very highly correlated (R^2^ = 0.994 to 0.999) for the recombinant human IL-10 provided with each kit. IL-10 concentrations were next analyzed in the vitreous samples from PIOL, OCL, and uveitis patients (Figure 2). Again, the two techniques produced good correlations (R^2^ = 0.701).

### Comparative Th1/Th2 cytokine profiles of PIOL, OCL, and uveitis samples

**Figure 3:**
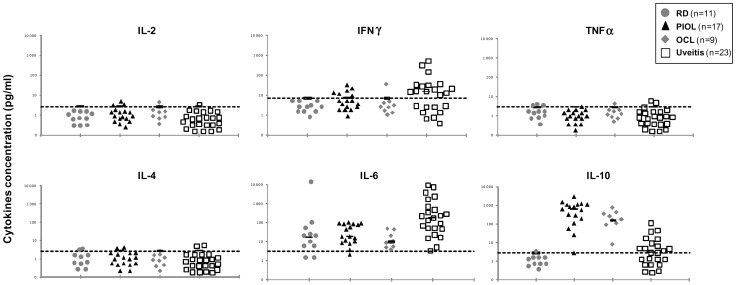
Th1/Th2 cytokine profile of RD, PIOL, OCL and uveitis samples. The gray circles represent the patients with retinal detachment (n = 11), the control group; the black triangles PIOL patients (n = 17), the gray diamonds OCL patients (n = 9), and the white squares uveitis patients (n = 23). The dashed lines indicate the sensitivity threshold for each cytokine: 2.6 for both IL-2 and IL-4, 2.8 for both IL-10 and TNFα, 3 for IL-6, and 7.1 pg/ml for IFNγ. All symbols represented under the sensitivity threshold had equivalent undetectable cytokine concentrations.

Vitreous samples from 60 patients (Table 1) with PIOL (n = 17), OCL (n = 9), uveitis (n = 23) or retinal detachment (RD) without hemorrhage (n = 11) were analyzed for IL-2, IL-4, IL-6, IL-10, IFNγ, and TNFα concentrations by CBA. IL-2, IL-4, and TNFα were detected only in a few samples and then with values near the threshold of detection (Figure 3). In the control RD samples, 82% (9/11) of cases had a significant level of IL-6, the only cytokine significant in this group. IL-6 was detected in 100% of the uveitis samples, IL-10 in 60%, and IFNγ in 48%. Interestingly, the PIOL and OCL cytokine profiles did not differ significantly, except for IL-10: the mean IL-10 concentration in PIOL samples was 724.12±676 pg/mL and in OCL samples, 247.41±207 pg/mL (*P* = 0.009). As previously described, the mean IL-10 concentration was much higher for PIOL (724.12±676 pg/mL) than for uveitis (12.61±23 pg/mL) samples (*P*<0.001). Inversely, the mean IL-6 concentration was lower for PIOL (41.4±35 pg/mL) than for uveitis (1184±2394 pg/mL) samples (*P* = 0.018). Nonetheless, some PIOL patients had low IL-10 levels and some uveitis patients high levels.

### IL-10/IL-6 and IL-10/IFNγ ratios for PIOL, OCL, and uveitis samples

**Figure 4:**
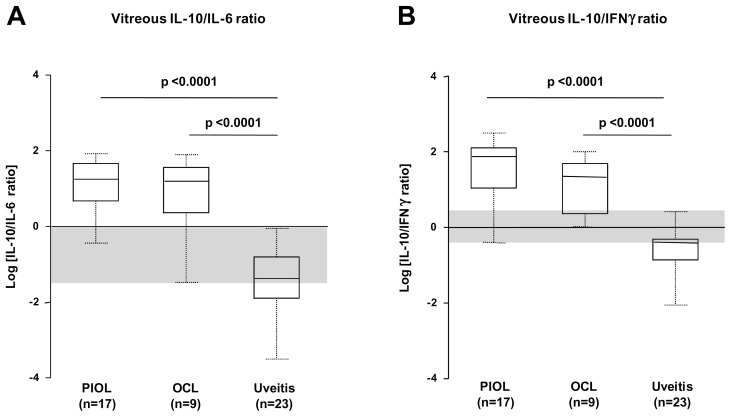
Vitreous IL-10/IL-6 and IL-10/IFNγ ratios for samples from PIOL, OCL, and uveitis patients. Box plots of the distribution of IL-10/IL-6 ratio (A), and IL-10/IFNγ ratio (B) for PIOL (n = 17), OCL (n = 9) and for uveitis (n = 23) patient groups. The upper and lower whiskers of each box represent the highest and lowest values, respectively, and the upper and lower borders of each box the 25^th^ (upper quartile) and 75^th^ percentiles (lower quartile). The line within each box is the median. The shaded region represents the “gray zone”, corresponding to an overlap of the range of values between the PIOL/PCL and uveitis groups, where diagnosis is uncertain.

Because IFNγ and IL-6 seemed to increase the IL-10 level of discrimination, we calculated IL-10/IL-6 and IL-10/IFNγ ratios to improve discrimination of PIOL/OCL versus uveitis patients (Figure 4). To display the ratio values above or below 1 proportionally on the graph (since, for example, a 2/1 ratio is 2 and a 1/2 ratio is 0.5), we decided to apply a Log_10_ transformation to all data (thus a 2/1 ratio is 0.301 and a 1/2 ratio −0.301) on a linear y-axis. When we applied the threshold level of detection for negative samples, we found mean IL-10/IL-6 and IL-10/IFNγ ratios higher both for PIOL (IL-10/IL-6, 27.2±24; IL-10/IFNγ, 85.98±81) and OCL (IL-10/IL-6, 26.68±25; IL-10/IFNγ, 33.58±30), than for uveitis (IL-10/IL-6, 0.15±0.22; IL-10/IFNγ, 0.54±0.66) samples. The median IL-10/IL-6 and IL-10/IFNγ ratios did not vary between PIOL and OCL samples.

Despite the significant differences in the median IL-10/IL-6 and IL-10/IFNγ ratios between PIOL/OCL and uveitis samples, the range of values between these groups overlapped. This overlapping, shown as a gray zone (Figure 4), did not allow us to define a clear cutoff for discriminating PIOL/OCL from uveitis patients.

**Figure 5:**
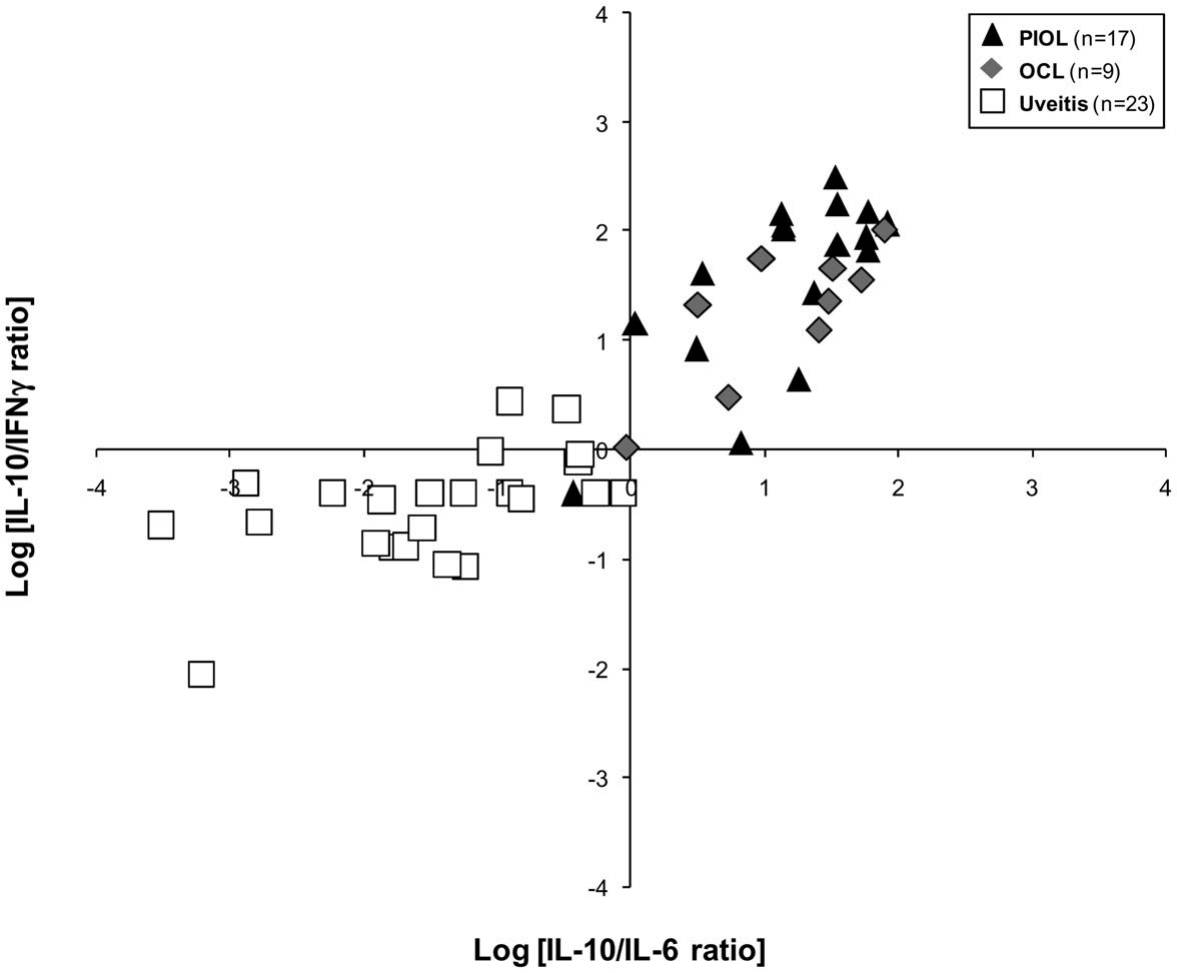
Combination of the Logarithmic conversion of IL-10/IL-6 ratio and IL-10/IFNγ ratios for vitreous samples from PIOL, OCL and uveitis patients. The black triangles represent PIOL patients (n = 17), the gray diamonds OCL patients (n = 9), and the white squares the uveitis patients (n = 23).

**Figure 6:**
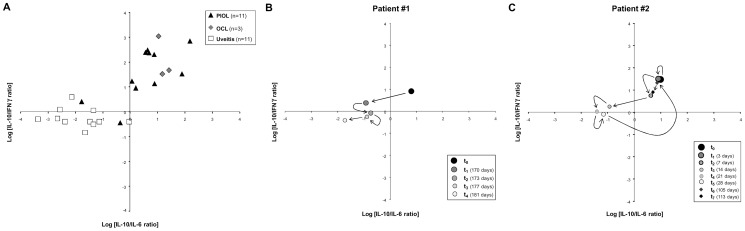
Combination of the Logarithmic conversion of IL-10/IL-6 and IL-10/IFNγ ratios in aqueous humor samples from PIOL, OCL and uveitis patients. (A) The black triangles represent PIOL patients (n = 11), the gray diamonds OCL patients (n = 3), and the white squares uveitis patients (n = 11). Kinetics follow-up after intravitreous methotrexate treatment of a remitting patient (B) and of a patient undergoing an initial phase of remission and then a relapse (C). For (B) and (C) size and gray intensity of the marks are correlated to the dates of analyses; the large black circle is the first time-point. Relapse is represented by black diamonds.

Since these overlapping samples were not necessarily for the same patient on each graph, we decided to combine the IL-10/IL-6 and IL-10/IFNγ ratios on a single graph (Figure 5). Using this strategy, we identified a cluster (upper right) containing exclusively PIOL and OCL samples. In the left parts of the graph, only 1 PIOL sample was colocalized with uveitis samples. We performed the same analysis on aqueous humor from PIOL, OCL, and uveitis patients and obtained similar results (Figure 6A). As aqueous humor can be harvested several times from the same patient, we were thus able to measure cytokine ratios over time during treatment (i.e., intravitreous injections of methotrexate). Remission was identified by a decrease in both ratios (see Figure 6B), whereas relapse was correlated with a new increase back in the upper right quadrant of the graph (Figure 6C).

## Discussion

The incidence of PCNSL has multiplied more than tenfold over the past decade. The incidence of PIOL, a subtype of PCNSL, has also increased albeit more slowly, thus leading to increased concern about the diagnosis of this disease [14], [15]. Moreover, PIOL is one of the most challenging masquerade syndromes, [5] especially for differential diagnosis with uveitis. The lack of information about this disease is mainly due to the limited number of patients diagnosed and the small amount of ocular fluid harvested. The principal reliable criterion for PIOL diagnosis is the presence of lymphoma cells in patient samples, but the cytological procedure is very delicate: lymphoma cells are particularly fragile ex vivo and sometimes difficult to find or to recognize; they can be analyzed only by highly trained hematologists. A high level of IL-10 in the vitreous of patients is also highly indicative of intraocular lymphoma but is not sufficient for a definitive conclusion [16]. This study describes the Th1/Th2 cytokine profile in undiluted vitreous and aqueous humor samples from PIOL patients, determined with a multiplex-based technology requiring only 25 to 50 µL of sample. First, we confirmed that IL-10 concentrations measured by CBA and ELISA were highly correlated, both for recombinant cytokine and human vitreous. Next, we observed that the results did not display either a conventional Th1 or Th2 profile: IL-2, IL-4 and TNFα were not detected in any sample. In the RD control group, the only cytokine detected at a significant level, and only in some samples, was IL-6. In contrast, as expected, samples from patients with PIOL and OCL both had high concentrations of IL-10; to a lesser extent, these samples also contained IL-6 and in some cases IFNγ. Interestingly, we noted that the cytokine profile of samples from patients with OCL at diagnosis was similar to that of samples from PIOL patients, except that the OCL patients had a slightly lower IL-10 concentration. Although the mean level of IL-6 and IL-10 expression in uveitis vitreous samples differentiated them from tumor samples, overlapping was found in some cases. Indeed, 48% (11/23) of the uveitis samples had an IL-6 concentration in the same range as PIOL/OCL samples and 38% (10/26) of the PIOL/OCL samples an IL-10 concentration in the same range as uveitis samples, consistent with a previous report [17]. Adding the IL-10/IFNγ ratio increased accuracy for PIOL diagnosis, but some samples still overlapped. Moreover, the exact cutoff ratio for a positive test result may vary between laboratories and by technique. The combination of the IL-10/IL-6 and IL-10/IFNγ ratios was highly informative in its discrimination between PIOL/OCL and uveitis samples. A PIOL/OCL specific cluster (100%, 25/25) was found and only 1 PIOL/OCL sample of the 26 (3.8%) was graphically colocalized with uveitis samples. In a recent paper, Ecker and colleagues [18] analyzed cytokines in the vitreous and aqueous humor of patients with posterior segment diseases and showed that the cytokine ratio was constant although cytokine quantities differed in the two locations. Similarly, we obtained the same cytokine ratios with aqueous humor as with vitreous. This is particularly important because aqueous humor is easier to tap and can be harvested several times during the disease. It could therefore be very useful for rapid prediction of patients' response to treatment, as reported recently by Kawamura and co-workers [19].

Accordingly, we recommend that ocular samples be frozen (−80°C) quickly in aliquots after harvesting. The IL-10/IL-6 and IL-10/IFNγ ratios in aqueous humor should then be routinely analyzed in aqueous humor from all patients with ocular inflammation at diagnosis and during treatment for PIOL/OCL, such as methotrexate and/or anti-CD20 monoclonal antibodies. We further encourage the use of multiplex technologies, such as cytometric beads arrays (CBA, BD Biosciences) that allow simultaneous measurement of several cytokines from a very low volume of liquid. Finally, the graphic representation of the combination of the Logarithmic (Log_10_) conversion of IL-10/IL-6 and IL-10/IFNγ ratios helps to cluster the PIOL/OCL versus uveitis patients at diagnosis, with the upper right cluster specific for PIOL/OCL patients.

In conclusion, the combination of the IL-10/IL-6 and IL-10/IFNγ ratios could be very helpful as an initial screening to rule out PIOL in uveitis patients. Whereas immunocytology and retinal histology remain the gold standard for confirming a diagnosis of PIOL, multiplex technologies are relatively simple to use for ruling this diagnosis out, they are accurate, and offer the opportunity to combine several markers for diagnosis and therapeutic follow up since only 25 to 50 µL of sample are needed to perform all these tests simultaneously.
